# Dynamics of Opposing Polymer Brushes: A Computer Simulation Study

**DOI:** 10.3390/polym13162758

**Published:** 2021-08-17

**Authors:** Krzysztof Hałagan, Michał Banaszak, Jarosław Jung, Piotr Polanowski, Andrzej Sikorski

**Affiliations:** 1 Department of Molecular Physics, Lodz University of Technology, Zeromskiego 116, 90924 Lodz, Poland; jaroslaw.jung@p.lodz.pl (J.J.); piotr.polanowski@p.lodz.pl (P.P.); 2 Faculty of Physics, Adam Mickiewicz University, Uniwersytetu Poznanskiego 2, 61614 Poznan, Poland; mbanasz@amu.edu.pl; 3 NanoBiomedical Centre, Adam Mickiewicz University, Wszechnicy Piastowskiej 3, 61614 Poznan, Poland; 4 Faculty of Chemistry, University of Warsaw, Pasteura 1, 02093 Warsaw, Poland; sikorski@chem.uw.edu.pl

**Keywords:** dynamic lattice liquid, lattice models, polymer brushes

## Abstract

Opposing polymer brush systems were synthesized and investigated by molecular modeling. Chains were restricted to a face-centered cubic lattice with the excluded volume interactions only. The system was confined between two parallel impenetrable walls, with the same number of chains grafted to each surface. The dynamic properties of such systems were studied by Monte Carlo simulations based on the dynamic lattice liquid model and using a highly efficient parallel machine ARUZ, which enabled the study of large systems and long timescales. The influence of the surface density and mean polymer length on the system dynamic was discussed. The self-diffusion coefficient of the solvent depended strongly on the degree of polymerization and on the polymer concentration. It was also shown that it is possible to capture changes in solvent mobility that can be attributed to the regions of different polymer densities.

## 1. Introduction

Polymer brushes are a system consisting of grafted macromolecules, i.e., chains terminally attached to a surface. They have been a subject of many experimental and theoretical works predominantly because of their practical importance (e.g., size-exclusion chromatography, polymer adhesion, or lubrication) [1,2,3,4]. Polymer brushes formed of chains grafted on one surface can be treated as the reference state for the confined brushes. Brushes were a subject of various experimental techniques of synthesizing, as recently reviewed [5,6,7]. Understanding the factors that govern the properties of a brush is therefore important for designing useful and intelligent polymeric systems [8]. Properties of brushes were studied by means of molecular dynamics and dissipative particle dynamics [9,10,11,12,13], Monte Carlo simulations [10,14,15,16,17,18,19,20,21,22,23,24,25,26,27], scaling theory, and self-consistent-field theoretical considerations [27,28,29,30,31,32,33,34,35].

Opposing polymer brushes, i.e., systems consisting of two parallel surfaces, both grafted with chains, were also a subject of considerable interest [36]. Theoretical treatment concerned mainly the compression of such brushes [37,38,39,40,41]. Computer simulations were found to be a useful tool for studying opposing polymer brushes—structure, interaction, and friction between a pair of brushes (neutral and charged) were recently investigated [39,42,43,44,45,46,47,48]. This study investigated the solvent and polymer dynamics for an opposing (sandwich-like) polymer brush, consisting of two flat surfaces on which the chains were grafted by polymerization. The brushes were synthesized using a ‘grafting from’ procedure; i.e., the polymerization of chains started from the surfaces. The dynamics of a formed opposing polymer brush was investigated focusing on the case when both brushed layers are in contact. It must be emphasized that a realistic but extremely small (from a computational point of view) probability of polymerization per algorithm time step (10^−6^) was used. A long relaxation time, up to 10^9^ time steps (total simulation time), showed to offer a new exciting opportunity to probe the equilibrium states of the confined dense polymer brushes. Because of the complex architecture, large size, and high density of polymer chains, these systems are usually studied employing coarse-grained models. The representation of macromolecules was highly reduced for this paper—chains consisted of interconnected statistical segments that were embedded to the nodes of a face-centered cubic lattice representing the simulated volume. Chains were firmly grafted to an impenetrable planar surface and a pair of them formed a slit. This model system was studied at good solvent conditions; i.e., all nonbonded interactions were the same in the whole system under consideration. Dynamic Monte Carlo simulations of the presented model were carried out using the dynamic lattice liquid (DLL) model dynamics [49,50]. This simulation algorithm provides the proper dynamics and reproduces the molecular transport in dense systems. DLL has already been successfully used, e.g., to study various polymerization processes [51,52,53].

## 2. Materials and Methods

The DLL model is based on the concept of strictly cooperative motion of objects in a dense system. The object refers here to the coarse-grained fragment of matter (polymer segment, solvent molecules, etc.). For simplicity, the positions of objects are limited to the nodes of a quasicrystalline lattice, in this case, FCC lattice with coordination number q = 12. It has been assumed that the system has some excess volume, so each object has enough space to vibrate around its position defined by the lattice node like in a real dense liquid. The objects, however, cannot easily move over a longer distance, because all neighboring lattice sites are occupied. Despite this, a long-range motion can occur as a cooperative rearrangement having a form of a closed loop of coordinated displacements involving at least three neighboring objects. In contrast to many other lattice models, DLL allows studying lattice systems at the highest density, i.e., where all lattice sites are occupied by exactly one object: a solvent molecule or a polymer segment. The DLL model described above has been implemented as a dynamic Monte Carlo simulation for polymer brushes in solvent. The single simulation time step t in the athermal case consisted of three steps:

1. The generation of random vector field of motion attempts. A unit vector, pointed towards one of the nearest neighboring lattice sites, represents the direction along which the object attempts to move;
2. The identification of groups of vectors forming closed loops, indicating ways of possible successful cooperative rearrangement. The rest of the objects are immobilized at the given time step. Additionally, if the movement realized in a given loop would lead to the break of a bond between segments in the polymer chain, then the loop is immobilized;
3. The rearrangement of objects along these closed paths by displacing them to the neighboring sites according to the vector generated in step 1.

A discussion concerning the detailed balance and ergodicity of DLL algorithm was presented elsewhere [49]. Time was assumed to be a discrete variable for which the positions of all objects were attempted to be updated simultaneously. As compared to experiments, one time step in DLL corresponds to 6·10^−13^ s for low-weight molecular systems [54] up to 3·10^−12^ s in the case of a polymer system [22].

In this paper, a coarse-grained model of multichain polymer systems forming opposing polymer brushes is under consideration. The system contained fully flexible chains immersed in a good solvent. The solvent was explicitly included in the model. The simulation procedure consisted of two steps. In the first step, polymer chains grafted on a pair of parallel surfaces were virtually synthesized. Note that polymer brushes in real experiments can be obtained using two different methods: by tethering the chains that were previously polymerized and by growing chains from initiators anchored to the surface [55,56,57,58]. The second one enables a highly grafted brush to be obtained, and what is more, in computer simulations, it is the only way to obtain properly equilibrated, highly grafted, and dense polymer systems [6,22]. Therefore, the second method was chosen for the presented studies. In the second step, the reaction was stopped and the system was fully equilibrated after the synthesis was completed. In this paper, the authors focus on the dynamic properties of the system after the synthesis part is finished.

## 3. Results

The system under consideration had a form of a slit built by a pair of parallel impenetrable surfaces placed at coordinates *z* = 1 and 144; i.e., the width of the slit was 2*d* = 142. The edge of the Monte Carlo box in each of the two remaining directions of the space had a length of *L* = 144; there were 1,492,992 FCC lattice nodes in total. Periodic boundary conditions were used in *x*- and *y*-direction. The large number of nodes was crucial for a good average necessary for the analysis of diffusion-type changes. The end of each chain was grafted (tethered) to one of the surfaces. Grafting positions were selected at random. The synthesized systems were polydisperse and, therefore, must be described by means of the averaged degree of polymerization (average chain length). The number averaged degree of polymerization *DP_n_* is defined as $DP_{n}=\sum_{i=1}^{2N}n_{i}m_{i}/\sum_{i=1}^{2N}n_{i}$, where 2*N* is the total number of chains in the system, *m_i_* is the length (number of segments) of ith chain, and *n_i_* is a number of chains with length *m_i_*.

The number of chains defines the grafting density of both brushes, which was defined as the number of chains grafted to one surface to the number of lattice nodes forming a surface. The grafting density was varied: *σ* = 0.2, 0.25, 0.3, 0.35, and 0.4 (for each surface). The studies concerning the influence of the grafting density were carried out for systems where *DP_n_* = 110, i.e., when both brushes interact. The overlap grafting density is usually defined as [11] σ* = π *R*^2^*_g_N*/*L*^2^, where *R*^2^*_g_* is the chain mean squared radius of gyration and *N* is the number of chains grafted to one of the surfaces. This parameter is a measure of the compression of grafted chains: for values σ* > 1, chains are restricted to less area (in the *xy* plane) than they would occupy in solution. In this study, σ* varied between 62 and 149, which implies that the chains under consideration were always in the real brush regime [4]. The grafting density *σ* = 0.3 was chosen for the investigation of *DP_n_* impact, based on previous studies concerning a model of a single brush [22]. The influence of the grafting density on the single brush structure has shown that a crossover from low to high grafting regime is located near this value. This value corresponds to 0.35 chains/nm^2^ in a polymer system when one polymer bead represents an MMA monomer unit [22]. For this grafting density, the mean number averaged degree of polymerization was varied in a wide range: *DP_n_* = 30, 50, 70, 90, 100, 110, 120, 140, and 160.

In the first part of the simulation, the entire simulation box was filled with monomer and the initiator was randomly placed on both surfaces with a given grafting density. For the model of polymer synthesis, the controlled living irreversible radical polymerization was chosen; i.e., the process of attachment of monomers to a growing chain was irreversible and a reaction rate *p* = 10^−6^ was assumed (this choice was based on our previous findings—see [22,59] for details). The polymer layer grew until it reached the desired *DP_n_* value, which lasted even up to 5 × 10^8^ time steps. The above criterion of polymerization termination leads to the generation of polydisperse systems, which makes the theoretical interpretation difficult but much better reflects the properties of real brush systems [22]. Then the reaction was stopped, and the unreacted monomer was replaced by an inert solvent of the same size as the monomer. The second step was a production run to collect uncorrelated data; it lasted 5 × 10^8^ time steps. The ‘grafting from’ procedure where chains grow very slowly was found to be an efficient tool for the simulation of dense brushes [22]. Faster ‘grafting from’ polymerization process requires very long equilibration [59], while attaching to the surface previously prepared whole is completely ineffective.

Having the number of objects under consideration significantly exceeding 10^6^, it is impossible to study such a number of time steps using a typical computer cluster or supercomputer running the DLL algorithm [60]. Therefore, the usage of the dedicated computing hardware such as ARUZ (Analyzer of Real Complex Systems—in Polish, *Analizator Rzeczywistych Układów Złożonych*) [60,61,62,63,64] is inevitable to study macromolecular systems in this timescale. ARUZ was designed and constructed using the TAUR technology (Technology of Real Systems Analyzers—in Polish, *Technologia Analizatorów Układów Rzeczywistych*) developed at Lodz University of Technology [65]. The machine is located in BioNanoPark Lodz (Poland). The device is composed of almost 26,000 reconfigurable field-programmable gate arrays (FPGAs) interconnected in a 3D network. ARUZ is a scalable, fully parallel data processing system equipped with low-latency communication channels, dedicated to the simulation of dense systems containing a large number of elements interacting locally. ARUZ can perform DLL simulations for systems with several millions of objects with, e.g., 10^9^ time steps performed in just a few days (vs. approx. 200 days on HPC node using multithreading [60]). The usage of ARUZ was indispensable to execute DLL simulations at this time range in reasonable computing time. This was the first time that this timescale was reached for the DLL algorithm for a simulation box of the described size. In summary, it was possible to study very large systems (>10^6^ objects) for the highest possible density (taking into consideration polymer and explicit solvent molecules) and for a long timescale (10^9^ steps). MD and DPD simulations of coarse-grained models cannot handle such calculations.

**Figure 1:**
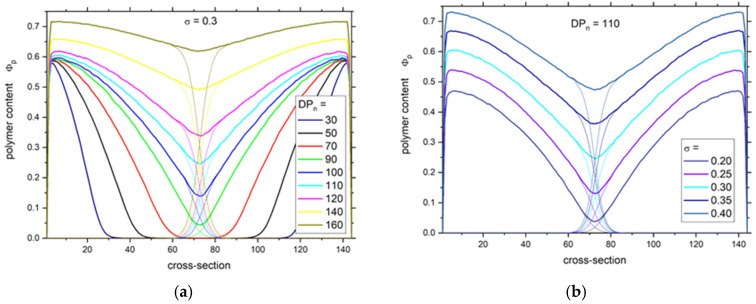
Polymer density profiles across the slit for different number averaged degrees of polymerization *DP_n_* (**a**) and for different grafting densities *σ* (**b**). Thick lines show total segment density (both brushes together) and thin lines represent the densities of single brushes.

The dynamics of complex systems like brushes is apparently connected to their internal structure and density. The polymer density profiles across a slit formed by a pair of grafted surfaces are presented in Figure 1. One can observe in Figure 1a that quite different polymer systems were under consideration: from two layers separated by a wide gap (ca. 40 lattice units) in the case of short chains (*DP_n_* = 30) to a system with an almost constant density of polymer beads across the slit (*DP_n_* = 160). This allows distinguishing two regions: (1) regions without brush interpenetration and (2) regions with interpenetration. The regions without interpenetration exhibit density profiles that are mostly linear, which agrees well with the SCFT calculations for polydisperse brushes. One can observe in Figure 1b that the increase in grafting density leads to higher density, but it does not change the shape of the density profiles.

**Figure 2:**
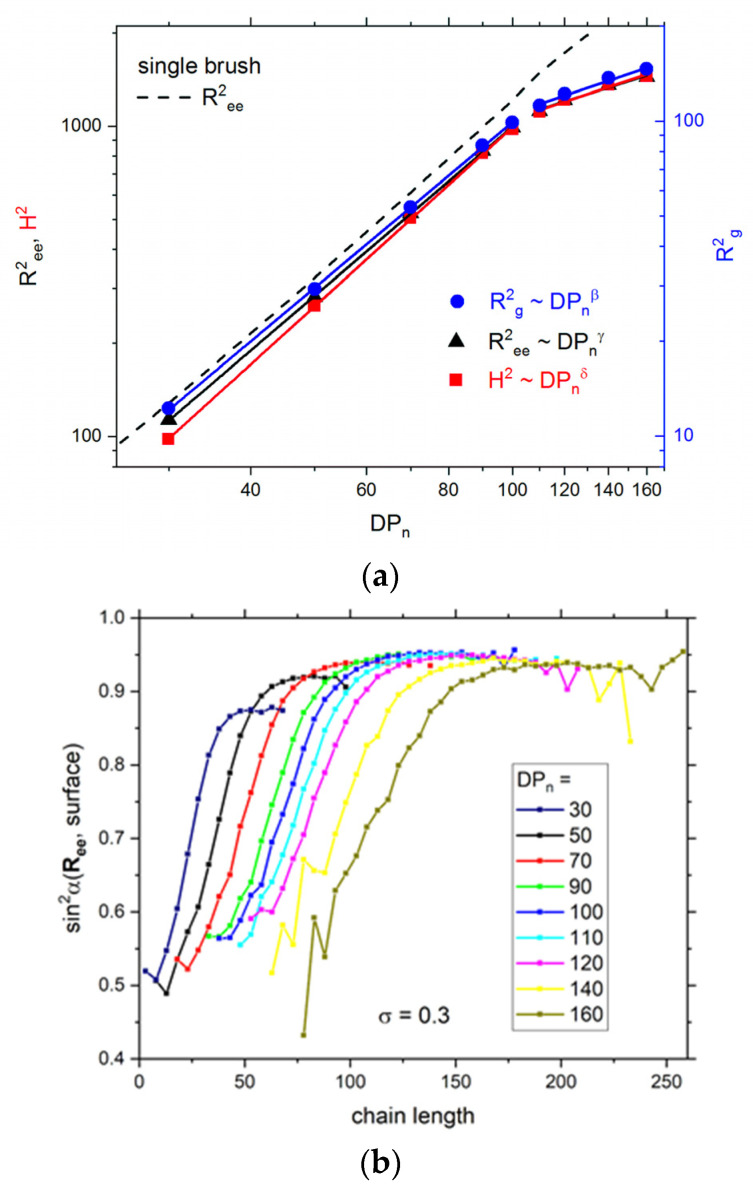
Mean squared end-to-end distance *R*^2^*_ee_*, mean squared height *H*^2^, and mean squared radius of gyration *R*^2^*_g_* as a function of *DP_n_*; the results for a single polymer brush are presented as a dashed line for comparison (based on simulations presented in [22]) (**a**). The angle between an end-to-end vector and the grafting surface (chains’ tilt angles) as a function of the chain length for different *DP_n_* values (**b**). The case of *σ* = 0.3.

The insight into the system structure can also be achieved by studying the scaling behavior of chain sizes. Three parameters describing the size of a single chain in a brush were considered here: the mean squared end-to-end distance *R*^2^*_ee_*, the mean squared height of a single polymer chain (*z*-component of a distance between the free end of a chain and the grafted surface) *H*^2^, and the mean squared radius of gyration *R*^2^*_g_*. The dependence of these parameters on *DP_n_*, which is a measure of the mean chain length, is presented as a log–log plot in Figure 2a. One can distinguish two regimes of the behavior of all size parameters, both characterized by a quite regular scaling behavior of *R*^2^*_ee_*, *H*^2^, and *R*^2^*_g_*. The first region includes chains with *DP_n_* ≤ 100, while the second one is observed for longer chains. The scaling behavior observed for double brushes consisting of shorter chains was found to be in the following form: *R*^2^*_ee_* ~ *DP_n_*^1.81±0.01^, *H*^2^ ~ *DP_n_*^1.92±0.02,^ and *R*^2^*_g_* ~ *DP_n_*^1.75±0.02^. The scaling exponents had similar values for all parameters and were much higher than in the dense polymer melt where 2*ν* ≈ 1 and than in the universal one describing the behavior of a single free chain, i.e., 2*ν* ≈ 1.176 [66]. They were also higher than predicted for the strongly stretched brush, where the exponent 1.5 was found from theoretical considerations and confirmed via off-lattice Monte Carlo simulations of living polymer brushes [27]. One must bear in mind that in the latter work grafting density was lower and the kinetics of growing chains was different (reversible polymerization). Thus, the main contribution to the higher values of exponents is the extension of chains along the normal to the grafting surface. The exponents concerning chains in the double brush are smaller than those for the fully extended chains (rods) where 2*ν* ≈ 2 and smaller than the exponents obtained by the bond fluctuation model where *ν* = 1.16 was found for grafting density 0.03 and 2*ν* = 1.93 was found for grafting density 0.1 [67]. In the second regime, i.e., for longer chains, the scaling exponents *R*^2^*_ee_* ~ *DP_n_*^0.67±0.07^, *H*^2^ ~ *DP_n_*^0.70±0.07^, and *R*^2^*_g_* ~ *DP_n_*^0.70±0.06^ are considerably lower than the exponent for dense polymer melt and even lower than for chains collapsed into globules, where 2*ν* ≈ 2/3. Moreover, one has to remember that the local polymer concentration is not very high and even for longer chains is between 0.3 and 0.7. This unexpected scaling behavior in the second regime can be explained by the increase in the size of short chains with increasing *DP_n_* and compression of longer ones resulting in flower conformation. The above behavior suggests that the mutual interaction of both brushes starts for chains *DP_n_* > 100, but it does also for shorter chains, as will be shown later. The conclusions drawn from the above discussion on the dependency of the chain size parameters vs. their length can be supported by the analysis of chain orientations. For this purpose, the angle between the end-to-end vector *R_ee_* and the grafting surface (the one to which the chain is grafted) was calculated [42]. Figure 2b presents the squared sinus of this angle as a function of the chain length. It is clear that the shortest chains exhibit smaller tilt angles, although these angles increase rather rapidly. The lowest values of sin^2^ are considerably above the mean value (1/3) that characterizes a random distribution of orientations. Therefore, the orientation of short chains can be treated as almost random regardless of the number averaged degree of polymerization *DP_n_* of the given system. For intermediate chains (length between 75 and 150 going from *DP_n_* = 50 to *DP_n_* = 160), tilt angles stabilize near the value 0.9; i.e., the longer chains are almost perpendicular to the grafting surface. The further increase in chain length leads to a slight decrease in tilt angles, apparently due to the impact of the second brush. What is interesting for long chains is that there is no difference in tilt angles for different values of *DP_n_* although both brushes are compressed.

**Figure 3:**
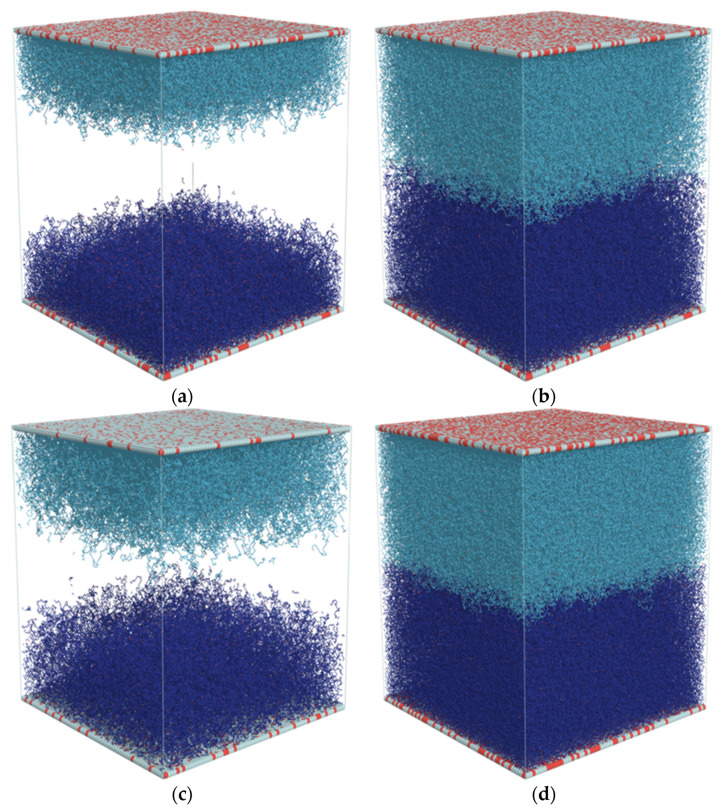
Configuration of an opposing polymer brush system for various values of *DP_n_* and grafting density *σ*. Each brush is displayed in a different color. Red dots mark ends of the polymer chains. *σ* = 0.1 grafting density was shown to present that in this case the brushes are not in contact. The cases of *σ* = 0.3, *DP_n_* = 50 (**a**); *σ* = 0.3, *DP_n_* = 110 (**b**); *σ* = 0.1, *DP_n_* = 110 (**c**); and *σ* = 0.4, *DP_n_* = 110 (**d**).

Figure 3a–d shows snapshots of the entire system under consideration for *DP_n_* = 50 and 110 and for *σ* = 0.1, 0.3, and 0.4. Solvent molecules are not shown for clarity. As each brush is marked with a different color, one can easily notice the border between them. One can observe that the increase in chain length leads to a very weak interpenetration of brushes. A similar situation occurred when the grafting density increases above *σ* = 0.3.

**Figure 4:**
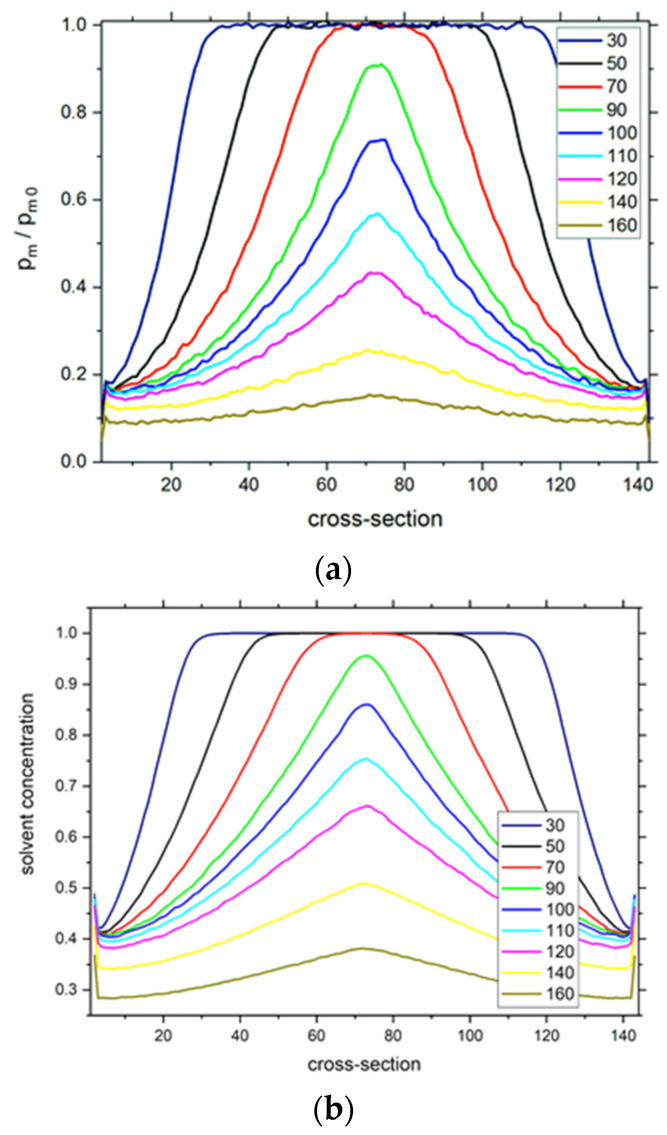
The normalized solvent mobility *p_m_*/*p_m_*_0_ as a function of the position across the slit (**a**). Solvent density profiles (**b**). The degrees of polymerization *DP_n_* are given in the insets. The case of *σ* = 0.3.

The dynamic properties of the solvent are discussed as a first point of the dynamic properties of the simulated system. The mobility of solvent was calculated as the probability of motion *p_m_* of a solvent molecule, i.e., the ratio of the number of performed moves in a given lattice node (when it was occupied by solvent) and the total time units in a simulation production run. This probability was averaged over the given plane *xy*. The probability of motion is connected to the movement waiting time, and a detailed discussion on these issues was already presented elsewhere [50,68]. In Figure 4 the reduced mobility is presented, i.e., mobility divided by the mobility calculated for solvent molecules in the solution without the presence of polymer chains *p_m_*_0_ = 0.0588 [68]. Figure 4 presents the changes in the reduced mobility of solvent molecules in the brush across the slit for different degrees of polymerization *DP_n_*. One can observe that the shapes of curves are almost opposite to those of the polymer density profiles presented in Figure 1. Significant changes in the probability of motion across the slit prove the heterogeneity of the system studied. The reduction in the solvent mobility is of an order of magnitude across the whole slit in the system with the highest degree of polymerization (*DP_n_* = 160). For opposing polymer brushes consisting of shorter chains, the reduction in *p_m_* is almost the same but only in the neighborhood of the grafting surfaces.

Figure 4b shows the solvent density profiles for the systems presented in Figure 4a. One can observe that the shapes of these curves are almost exactly the same as those of solvent reduced mobility and, thus, can be directly correlated.

**Figure 5:**
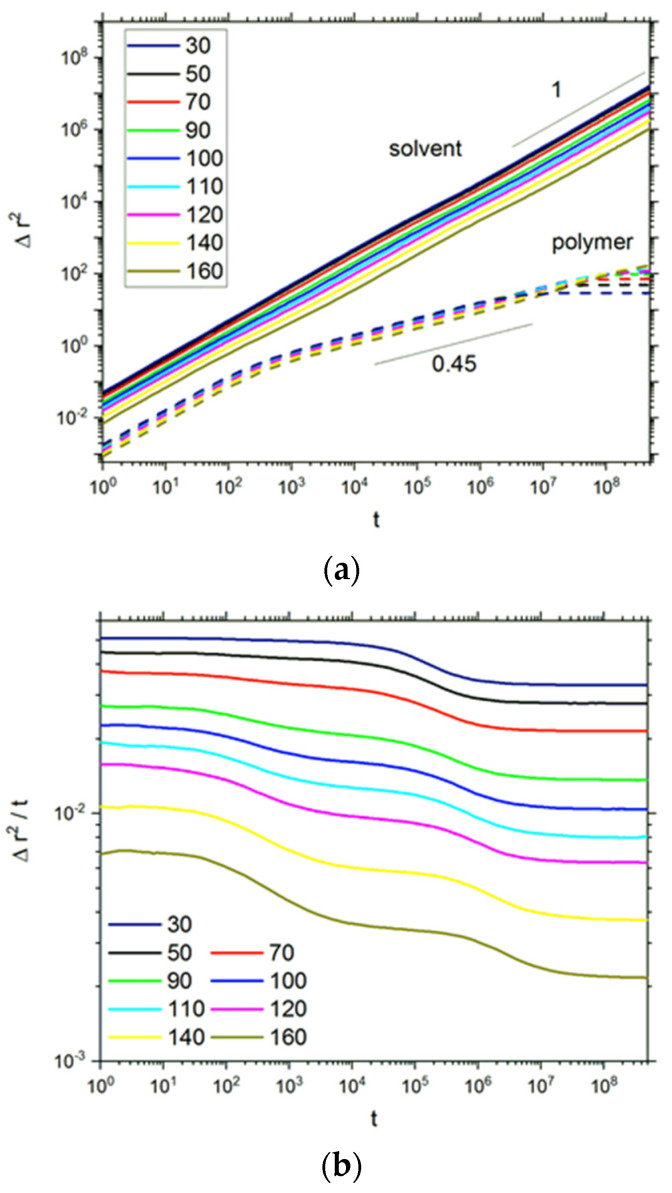

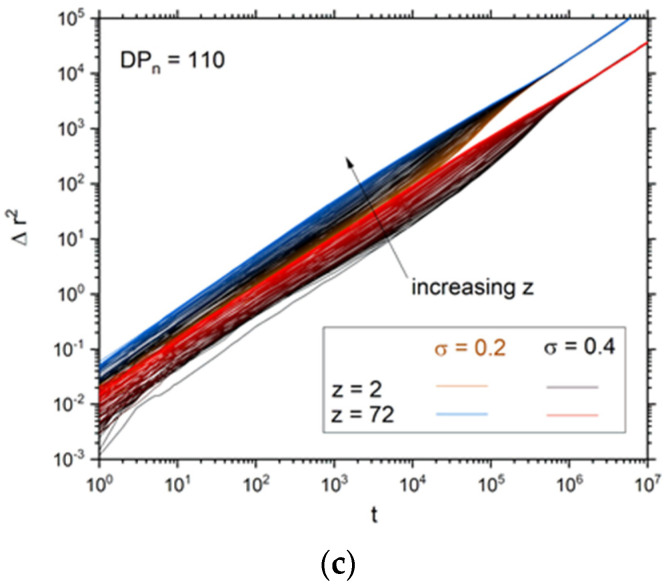
The mean squared displacement (MSD) of solvent and polymer molecules (**a**) and MSD/*t* (**b**) as functions of time for solvent. The degrees of polymerization *DP_n_* are given in the insets. The case of *σ* = 0.3. MSD for solvent with various initial surface proximities (cross-section number *z*) for two selected grafting densities (**c**).

The long-time dynamic properties of soft matter systems can be studied by means of mean squared displacement (MSD) Δ*r*^2^. The MSD of solvent molecules is defined as $\Delta r^{2}\left(t\right)=\frac{1}{N}\sum_{i=1}^{N}\left[{\left(r_{i}\left(t\right)-r_{i}\left(0\right)\right)}^{2}\right]$, where *r_i_*(*t*) are the coordinates of the *i*th bead at time *t* and *N* is the number of solvent molecules. In general, the dependency of the mean squared displacement on time can be written as Δ*r*^2^ ~ *t^α^*. If the diffusion is normal, i.e., it follows the Einstein relation with the exponent *α* = 1, the case *α* < 1 corresponds to a subdiffusive (anomalous) motion that is expected in complex macromolecular systems [69]. Figure 5a presents the MSD as a function of time in a double logarithmic plot. It seems that the plots for all brushes studied exhibit a common scaling behavior *t*^1^ for solvent, but the closer examination of these curves reveals nonlinearity, at least for intermediate times. A more detailed discussion of polymer dynamics is presented later. The mean squared displacement divided by time as a function of time was plotted (Figure 5b) to reveal a deviation from Einstein law and the appearance of anomalous diffusion for solvent. Here the MSD function parallel to the time axis corresponds to the case of normal diffusion. Such regions where a normal diffusion is present are clearly observed for a very short time and the end of the trajectory. At intermediate times, a subdiffusive motion appears for brushes with both low and high *DP_n_*. Except for brushes with *DP_n_* = 30–70, the shape of curves is more complex, and this effect is discussed below. The density of polymer in the system is apparently above the static percolation threshold, at least for brushes with a higher degree of polymerization. Despite this, the motion of solvent is not limited at a longer time, which can be explained by the fact that the obstacles, i.e., polymer chains, are also mobile—it was shown that the percolation threshold is not observed for mobile obstacles [70,71]. Further insight into anomalous diffusion can be obtained from the analysis of the solvent mobility in a given layer, i.e., at a given distance from the grafting surface (the closer one). Of course, during the simulation, solvent molecules can change layers; therefore, these results can be treated only as a qualitative description of the influence of the local structure of the system on solvent motion. Figure 5c presents the mean square displacement calculated for the solvent molecules that were located, at the beginning of the simulation, on the surface (*z* = 2) and in the middle of the slit (*z* = 72). The results presented concern the degree of polymerization *DP_n_* = 110, i.e., the case where opposed brushes interact with each other. The difference in polymer density is approximately 2-fold for these cases (see Figure 1a,b). Solvent molecules that start to move in the layers close to the surface are considerably hindered by the presence of polymer chains, and their mobility is an order of magnitude lower than in the middle of the slit. Moreover, a well-defined transient subdiffusive region is observed, and it is the deviation from the normal diffusion that decreases with the distance from the grafting surface. These results are consistent with the molecular dynamics simulations of atomistic and coarse-grained model polymers in the slit [72,73].

**Figure 6:**
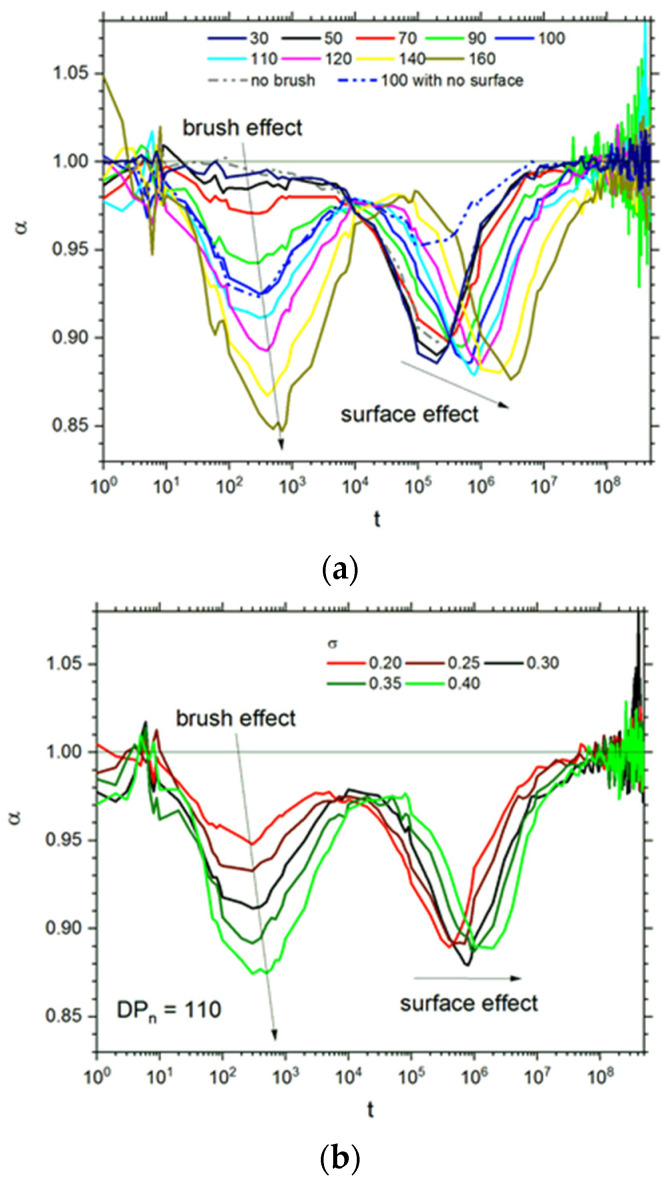
The exponent *α* (see text for details) as a function of time for various chain lengths at *σ* = 0.3 (**a**). Dashed lines represent the case of flat surfaces only (no chains) and surfaces without nongrafting surface sites (grafting chain ends were still immobile). The exponent *α* as a function of grafting density for *DP_n_* = 110 (**b**). The degrees of polymerization *DP_n_* and *σ* values are given in the insets.

It is difficult to identify regions of anomalous diffusion using only MSD function because the changes in the exponent *α* are rather small. Therefore, the exponent *α* was calculated as a logarithmic derivative $\alpha =d\left(log\left(\Delta r^{2}\left(t\right)\right)\right)/d\left(log(t\right))$. Figure 6a presents the dependency of the exponent *α* on time for *σ* = 0.3. The deviations from the value *α* = 1 were found at a time between 10^1^ and 10^4^ (the first minimum) and for considerably longer times between 10^4^ and 10^8^ (the second minimum). The depth of the first one approaches the value 0.85 for the highest degree of polymerization and shifts slightly towards longer times with the increase in *DP_n_*. The same behavior was observed for the second minimum, although the changes in depth were smaller while the shift was considerably larger. To recognize the reason for the appearance of these two deviations from the normal diffusion, additional simulations were carried out: for an opposing polymer brush without grafting surfaces (the ends of chains were pinned and located at a virtual surface) and for a slit filled with solvent molecules but without polymer chains. The results of these additional simulations are included in Figure 6a. The exponent *α* calculated for a slit with solvent only does not exhibit the first minimum and does exhibit the second one. This behavior implies that the first minimum relates to the presence of an opposing polymer brush in the slit, while the second one is apparently caused by the presence of a pair of impenetrable surfaces. The confirmation of this statement can be found by analysis of the behavior of *α* for an analogous polymer system but without surfaces. Here the first minimum is observed (although the second one is also present but considerably shallowed—grafting sites are still present and immobile). Minima on the exponent *α* curves for polymer solutions without confining surfaces were also found for a time near 10^3^ (these curves, however, were less complex) [74].

Figure 6b presents the changes in the exponent *α* with time for various grafting densities *σ*. The *σ* value does not influence the shape of *α*(*t*) curves, and the increase in *σ* shifts the minima of curves towards longer times. The changes in *α* depend strongly on the grafting density: the higher *σ* is, the deeper the minimum on an *α* curve is. The depth of the second minima, i.e., the one caused by the presence of the walls, does not change.

**Figure 7:**
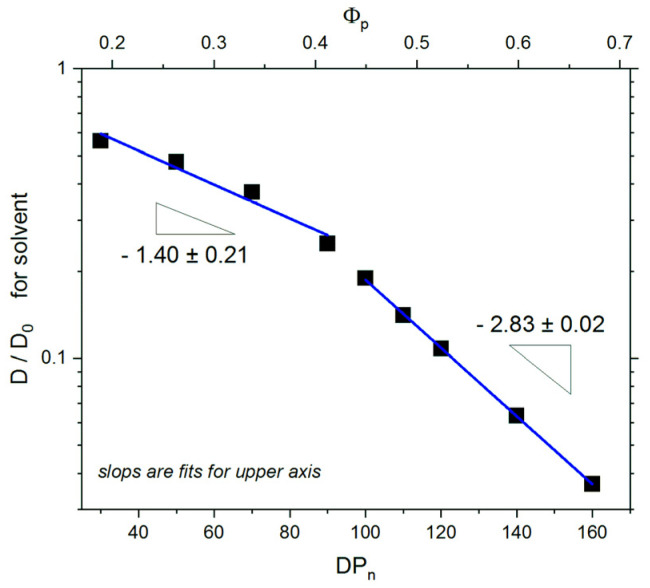
Reduced self-diffusion coefficient *D*/*D*_0_ for solvent as a function of the degree of polymerization *DP_n_*. The case of *σ* = 0.3.

Long-timescale dynamics of the system is usually characterized by the self-diffusion coefficient *D* calculated from the mean squared displacement Δ*r*^2^ as *D* = Δ*r*^2^/6*t*. Values of the diffusion coefficient were determined in time windows where the diffusion was Fickian, i.e., where Δ*r*^2^ ~ *t*^1^. Such regions of MSD were found for all systems under consideration at the longest times, i.e., at the ends of trajectories. Figure 7 presents the self-diffusion coefficient *D*/*D*_0_ as a function of the degree of polymerization, normalized by the value determined for a system containing molecules of solvent only with no polymer and no surfaces. One can easily identify two regimes of the self-diffusion coefficient scaling behavior. In both regimes, the ratio *D*/*D*_0_ decreases linearly: for a small degree of polymerization (*DP_n_* ≤ 90), where brushes are mostly separated, *D*/*D*_0_ ~ *DP_n_*^−1.40^. For compressed brush systems with a higher degree of polymerization, this dependency was found considerably stronger: *D*/*D*_0_ ~ *DP_n_*^−2.83^. One has to remember that the degree of polymerization is proportional to the polymer concentration according to the formula $\Phi_{p}=2n\langle DP_{n}\rangle /\left(2dL^{2}\right)$. Therefore, the dependence on the polymer concentration can be simultaneously studied.

**Figure 8:**
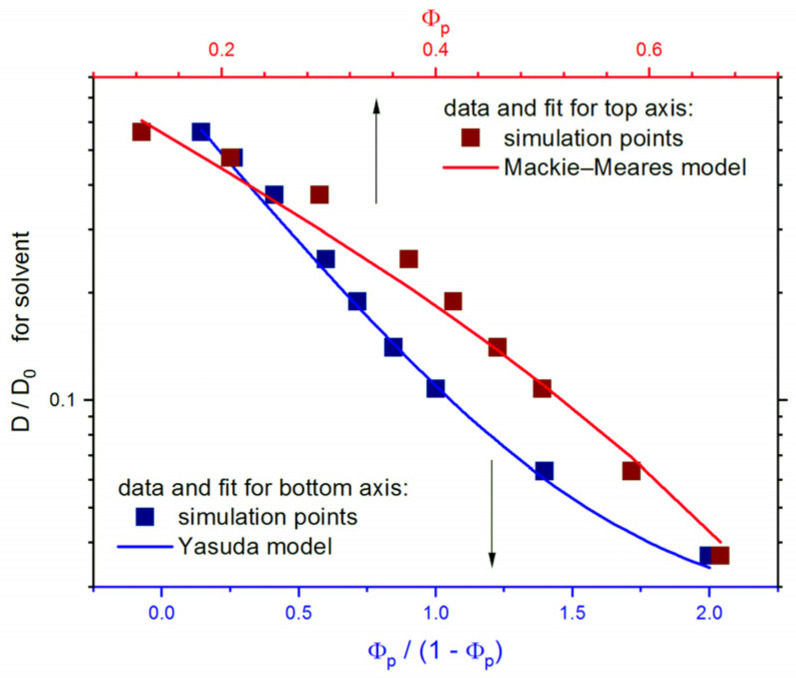
Solvent reduced self-diffusion coefficient *D/D*_0_ as a function of the polymer concentration *Φ_p_* (top axis) and *Φ_p_* /(1 − *Φ_p_*) (bottom axis). The fits to Mackie–Meares and Yasuda theories are also marked. The case of *σ* = 0.3.

One could also approximate the changes in the reduced self-diffusion coefficient by formulas determined from theoretical considerations [75]. Two simple theories have been chosen. The first was the Mackie–Meares [76]; as one of the obstructive theories (based on the probability of occupation of neighboring lattice sites), it seems to be the most appropriate because of the model used in simulations *D*/*D*_0_ = ((1 − *Φ_p_*)/(1 + *Φ_p_*))^2^. The Yasuda theory [77] was also applied. This theory, based on free volume (an effective free volume is attributed mainly to solvent molecules), was used because it turned out useful for polymer systems studied by means of the DLL model [54,74] *D*/*D*_0_ = exp(*BΦ_p_*/(1 − *Φ_p_*)), where *B* is a constant depending on the free volume. Figure 8 presents the reduced self-diffusion coefficient *D*/*D*_0_ as a function of polymer concentration *Φ_p_* and *Φ_p_* /(1 − *Φ_p_*) and the results of the fits to both above-mentioned theories. One can observe that both fits are quite good (especially for high *Φ_p_*), but the Yasuda theory gives a slightly better approximation. It has to be remembered that the opposing polymer brushes studied in this work, except for the systems characterized by a very high degree of polymerization, are systems that are not homogeneous with respect to polymer concentration. Therefore, despite a good fit, the better choice is to describe the changes in the self-diffusion coefficient by scaling relations as presented above in Figure 7, where differences between the two types of brushes are clearly visible. The dynamics of solvent in opposing polymer brushes can also be compared to the diffusion of solvent molecules in other polymer systems studied within the frame of the DLL model, although one has to remember that those studies dealt with monodisperse systems [54,74]. In two-dimensional solutions containing macromolecules with frozen conformations, *D*/*D*_0_ behaves in a different way; i.e., it follows a modified version of Mackie–Meares and Yasuda theories (the modification was necessary due to the percolation problem not present here) [57]. In three-dimensional systems, in a wide range of polymer lengths, the scaling behavior of *D*/*D*_0_ was found, and the scaling exponent was calculated as 1.34, i.e., slightly lower than that for separated opposing polymer brushes studied here [74].

**Figure 9:**
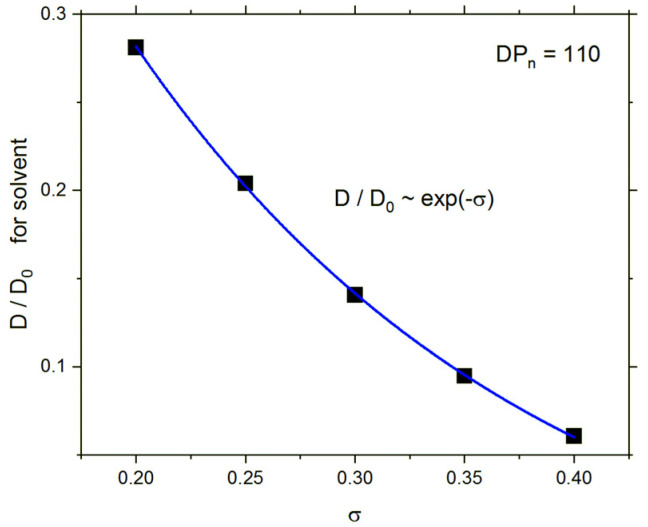
Solvent reduced self-diffusion coefficient *D*/*D*_0_ as a function of grafting density *σ*.

The influence of the grafting density on the reduced self-diffusion is presented in Figure 9. The decrease in solvent mobility is strong, and one can describe it as exponential: *D*/*D*_0_ ~ exp(−*σ*).

**Figure 10:**
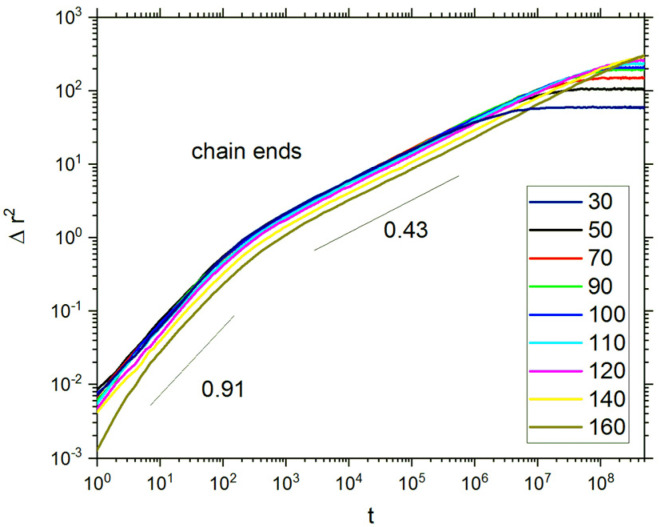
Mean squared displacement of chain ends as a function of time. The degrees of polymerization *DP_n_* are given in the inset. The case of *σ* = 0.3.

The dynamics of macromolecules in opposing polymer brush systems is also interesting as the motion of solvent molecules occurs in cooperation with polymer beads. The motion of the entire macromolecules is restricted due to their grafting, and therefore, the motion of chain ends, which are the most mobile, was studied. It should be noted that higher mobility of chain ends (when compared to inner polymer beads) was also found for free macromolecules in bulk [78,79]. It is shown that the upper part of the grafted chain relaxes an order of magnitude faster than the part close to the grafting point [42,43]. Figure 10 presents the mean squared displacement of chain ends as a function of time in a double logarithmic plot. Three different regimes can be distinguished for each degree of polymerization. In each regime, all chains exhibit the same scaling behavior regardless the degree of polymerization: the short time regime (below 10^2^), where Δ*r*^2^ ~ *t*^0.91^; the intermediate regime (between 10^2^ and 10^6^), where Δ*r*^2^ ~ *t*^0.43^; and the long time regime, where Δ*r*^2^ remains constant. The last regime corresponds to a limited motion, which is obvious as the chains are firmly anchored to the grafting surfaces. Using the same DLL model for a solution of polymer chains that can freely move (systems with varied chain lengths and concentrations but monodisperse), a different scaling was found: roughly Δ*r*^2^ ~ *t*^1^ for short times (<10^2^) and Δ*r*^2^ ~ *t*^0.3^ (short chains) and *t*^0.4^ (long chains) for longer times (between 10^2^ and 10^5^) [54]. Recent Monte Carlo simulation studies of single brushes based on the bond fluctuation model and with the grafting density *σ* = 0.11 showed that the mean squared displacement scales with time like *t*^0.5^ (at longer time) and then flattens out [80].

## 4. Discussion

Dynamics of opposing polymer brushes were studied using a unique simulation algorithm dynamic lattice liquid (DLL) model based on the cooperative movement concept and unique hardware Analyzer of Real Complex Systems (ARUZ). DLL is a class of Monte Carlo simulation algorithm. In this model, cooperative rearrangements of a system have the form of closed loops of displacements, and this model allows the study of lattice systems (face-centered cubic lattice in this work) with all lattice sites of the systems occupied by polymers and solvent molecules. ARUZ is a fully parallel data processing system equipped with low-latency communication channels, dedicated to the simulation of systems consisting of a large number of elements interacting locally. The polymerization process in which opposing polymer brush systems were obtained was performed by means of DLL with realistic reaction parameters. In summary, opposing polymer brushes were simulated with a variety of enhancements at once, such as large system size, long simulation times, high grafting density, high polymer concentration, and realistic polymerization using the DLL algorithm on the state-of-the-art ARUZ hardware.

The main conclusions of this paper are related to the closely connected structural and dynamic properties of opposing polymer brushes. Main results indicated that the density profiles of unconstrained and weakly constrained brushes were almost linear functions of distance (except edges) from the surfaces. This kind of behavior is expected for polydisperse brushes and therefore justifies the choice of method. The focus was put on the motion of solvent molecules in such a complex system as studied. The appearance of anomalous diffusion for all systems studied was shown; what is more, the short- and long-time diffusion was found to be normal, satisfying Einstein’s law. The mobility of solvent depended on the distance from the grafting surface and reflected polymer density profiles. It was also shown that the long-time self-diffusion coefficient depended strongly on the degree of polymerization and on the polymer concentration. This dependency was found more pronounced for brushes consisting of longer chains. The changes in the self-diffusion coefficient with polymer concentration showed that it can be described by obstructive or free volume theories and, generally, by simple scaling relations with high scaling exponent. Based on the mobility of solvent in different layers of the slit, the possibility to capture the changes in solvent mobility was shown. It can be attributed to the escape of solvent from a dense polymer system into a more mobile solvent region—this was possible for opposing polymer brushes with a well-defined gap between them.

## Data Availability

The data that support the findings of this study are available from the corresponding author upon reasonable request.
